# Intact Lexicon Running Slowly – Prolonged Response Latencies in Patients with Subthalamic DBS and Verbal Fluency Deficits

**DOI:** 10.1371/journal.pone.0079247

**Published:** 2013-11-13

**Authors:** Felicitas Ehlen, Lea K. Krugel, Isabelle Vonberg, Thomas Schoenecker, Andrea A. Kühn, Fabian Klostermann

**Affiliations:** 1 Department of Neurology, Motor and Cognition Group, Charité – University Medicine Berlin, Berlin, Germany; 2 Department of Neurology, Charité – University Medicine Berlin, Berlin, Germany; Charité University Medicine Berlin, Germany

## Abstract

**Background:**

Verbal Fluency is reduced in patients with Parkinson’s disease, particularly if treated with deep brain stimulation. This deficit could arise from general factors, such as reduced working speed or from dysfunctions in specific lexical domains.

**Objective:**

To test whether DBS-associated Verbal Fluency deficits are accompanied by changed dynamics of word processing.

**Methods:**

21 Parkinson’s disease patients with and 26 without deep brain stimulation of the subthalamic nucleus as well as 19 healthy controls participated in the study. They engaged in Verbal Fluency and (primed) Lexical Decision Tasks, testing phonemic and semantic word production and processing time. Most patients performed the experiments twice, ON and OFF stimulation or, respectively, dopaminergic drugs.

**Results:**

Patients generally produced abnormally few words in the Verbal Fluency Task. This deficit was more severe in patients with deep brain stimulation who additionally showed prolonged response latencies in the Lexical Decision Task. Slowing was independent of semantic and phonemic word priming. No significant changes of performance accuracy were obtained. The results were independent from the treatment ON or OFF conditions.

**Conclusion:**

Low word production in patients with deep brain stimulation was accompanied by prolonged latencies for lexical decisions. No indication was found that the latter slowing was due to specific lexical dysfunctions, so that it probably reflects a general reduction of cognitive working speed, also evident on the level of Verbal Fluency. The described abnormalities seem to reflect subtle sequelae of the surgical procedure for deep brain stimulation rather than of the proper neurostimulation.

## Introduction

Deep brain stimulation (DBS) of the Subthalamic Nucleus (STN) is a well-established treatment for patients with advanced Parkinsońs disease (PD). It strongly suppresses motor symptoms and complications from long-term dopaminergic therapy [1]–[4]. Although relevant cognitive DBS sequels are altogether rare, declines in Verbal Fluency (VF) tasks have frequently been observed. [5]–[19]. Such tasks require participants to utter as many words as possible belonging to a certain category or starting with a predefined letter in a given period of time (usually 60 or 120 s). Testing VF typically involves frontal functions, such as working memory and set shifting, besides primary phonemic and semantic operations [20], [21]. In the particular case of DBS, VF deficits were therefore mostly categorised as dysexecutive [12], [22]–[24]. Neuroanatomically, they have rather been allocated to frontal lesions along the trajectory rather than to effects within the STN [5], [8], [19], [25]–[28], because in most studies they were found to occur postoperatively regardless of the stimulation state [25], [27], [29], [30].

However, various studies in patients with STN-DBS found no correlations between changes of VF and other ‘frontal’ functions [8], [15], [16], [25], [27], [30]–[32] and, interestingly, similar dissociations have been described in non-DBS PD patients [33]. Further, in a number of studies, mainly phonemic VF was found impaired in PD patients with DBS [5], [14], [17], [29], compatible with a dysfunction in a specific lexical domain.

It is of conceptual interest that different theories about the involvement of subcortical structures in linguistic functions have been formulated. In particular, the ‘Lexical Selection’ [34], [35] and ‘Response-Release Semantic Feedback’ [36]–[38] models posit that the basal ganglia participate in word recruitment and release, whereas the ‘Declarative/Procedural’ [39]–[42] and ‘Selective Engagement’ [43] models argue against a role of these structures for lexically-specific operations. Furthermore, time-critical (de-)coding of mental operations in general has been ascribed to the basal ganglia [44], [45].

In this context, Lexical Decision Tasks (LDTs) [46] could provide useful information. In LDTs, subjects have to differentiate word-nonword from word-word sequences. This differentiation is accelerated if two sequential words are semantically or phonemically related. Accordingly, latencies of word-nonword decisions reflect the overall speed of word processing, and their acceleration by phonemic or semantic priming mirrors process facilitation in lexical subdomains [47]–[51]. With respect to VF, LDT result patterns may thus demonstrate whether abnormalities are associated with particular lexical dysfunctions or with more general changes of processing speed.

So far, only one study investigated semantic priming in PD patients with active versus inactive DBS but addressed a different research question, suggesting a restoration of controlled processes by the stimulation. VF and phonemic priming were not tested along with semantic priming [22].

Against this background, we investigated the VF and LDT performance in PD patients treated with and without DBS. DBS and non-DBS patients were tested in ON and OFF DBS and, respectively, medication conditions. In so doing, we aimed to explore whether STN-DBS or PD drugs exert an influence on VF performance that can be explained by altered lexical activation (indicated by priming effects) or by more general changes in cognitive speed (indicated by the overall reaction time). To evaluate possible interactions with the disease itself, a group of age matched healthy controls also participated in the trial. The findings are discussed under a conceptual and clinical view.

## Methods

### Ethics Statement

All subjects gave written informed consent to the study protocol approved by the ethics committee II of the Charité (protocol number EA2/047/10).

### Participants

66 subjects took part in this study. 47 suffered from PD, either treated only with antiparkinsonian medication (non-DBS group, n = 26) or additionally with bilateral STN-DBS (DBS group, n = 21). 19 healthy persons participated as age matched controls (for details see Table 1).

**Table 1:** Overview of participantś baseline characteristics.

	Cntr	PD group		*p-values*		
		non-DBS	DBS	Controls vs.non-DBS	Controls vs.DBS	non-DBS vs.DBS
	*n = 19*	*n = 26*	*n = 21*			
		*[n = 18]*	*[n = 19]*			
Characteristics						
	M (± SD)	M (± SD)	M (± SD)			
Age (years)	67.2 (±8.1)	67.3 (±8.2)	64.7 (±8.6)	.97	.35	.30
		[65.5 (±8.5)]	[65.2 (±8.0)]	[±.54]	[±.44]	[±.90]
Educ. (years)	10.7 (±1.9)	10.7 (±1.6)	10.2 (±1.5)	.88	.36	.38
		[10.8 (±1.7)]	[10.4 (±1.5)]	[±.38]	[±1.00]	[±.38]
Gender (f/m)	7/12	13/13	4/17	.38	.21	.03
		[9/9]	[4/15]	[±.42]	[±.28]	[±.07]
Handedness (r/l)	16/3	24/2	20/1	.39	.25	0.68
		[16/2]	[18/1]	[±.68]	[±.29]	[±.52]
net PANDA	19.5 (±3.1)	18.0 (±3.1)	17.5 (±3.3)	.17	.06	.65
(points)		[18.6 (±3.6)]	[17.5 (±3.4)]	[±.33]	[±1.00]	[±.33]
Disease duration		11.2 (±6.2)	14.1 (±4.5)			.07
(years)		[14.5 (±4.0)]	[15.1 (±3.5)]			[±.63]
Side of onset		14/9/3	17/2/2			.11
(r/l/b)		[9/6/3]	[15/2/2]			[±.16]
LED (mg)		1147.4 (±755.6)	573.6 (±383.2)			.00
		[1341.9 (±788.1)]	[570.1 (±349.3)]			[±.00]
UPDRS III (points)		25.0 (±14.1)	19.8 (±7.1)			.13
		[27.2 (±15.5)]	[20.6 (±6.9)]			[±.10]
HY (stage)		2.1 (±.9)	2.6 (±.7)			.08
		[2.1 (±1.0)]	[2.6 (±.7)]			[.92]

*HY*: Hoehn & Yahr; *net PANDA:* Parkinson Neuropsychometric Dementia Assessment (PANDA) after subtraction of the test item Verbal Fluency (max. = 23 pt); *f*: female; *m*: male; *r*: right; *l*: left; *b*: bilaterally; *LED*: total daily levodopa equivalent dose; *UPDRS III*: Unified Parkinson’s Disease Rating Scale – motor score (max. = 108 pt).

Values indicate the mean (± standard deviation). Values in each upper row relate to each entire group of patients. Values in the lower rows, given in brackets, relate to the subgroup of long-term diseased patients. Disease related values refer to the treatment ON condition.

Patients were recruited from the Outpatient Clinic for Movement Disorders of the Charité and fulfilled the diagnostic Brain Bank Criteria for PD. They were excluded if diagnosed with brain diseases other than PD including all psychiatric disorders, such as depression, psychosis or apathy (according to the criteria of the German Manual for Psychopathological Diagnosis, AMDP [52]), if they scored below 15 points in the Parkinson Neuropsychometric Dementia Assessment (PANDA) [53] or had hearing problems interfering with task performance. All patients received either a monotherapy with levodopa (or in a few cases with a dopamine agonist) or levodopa in combination with other antiparkinsonian drugs such as entacapone, amantadine, or a dopamine agonists. All participants were native German speakers. The groups were matched for age, years of education, PANDA, and the motor score of the Unified Parkinson’s Disease Rating Scale (UPDRS) under therapy.

33 patients were tested ON versus OFF medication (14 subjects from the non-DBS group) or, respectively, stimulation (19 subjects from the DBS group). In the treatment OFF assessment one patient from the non-DBS group and three patients from the DBS group were not able to complete all VF tasks due to a generally reduced condition.

The interval between the two sessions was two months. The order for the examinations in either state was randomized. Medication OFF was defined as an overnight PD drug withdrawal of at least 12 hours. For the DBS OFF condition stimulation had to be switched off at least 30 min before experiments were started. ON states were defined as conditions under continued treatment with the current drug regime and, in the DBS group, under the therapeutic stimulation parameters (see Table 2). In the DBS group the current therapeutic drug regime was maintained in both sessions.

**Table 2:** Overview of DBS settings.

						Electrode coordinates				
		Frequency (Hz)	Amplitude (V)	Pulse width (µs)	TEED_1sec_	Polarity (mono/bi)	x (mm)	y (mm)	z (mm)	DBS duration (years)
P1	right	145.00	2.20	60.00	57.29	mono	–	–	–	12
	left	145.00	2.60	60.00	115.09	mono	–	–	–	
P2	right	90.00	2.90	60.00	39.66	bi	12.07	−14.98	−7.62	2
	left	90.00	3.40	90.00	114.47	mono	11.85	−13.13	−8.88	
P3	right	130.00	2.60	60.00	69.84	mono	12.26	−14.19	−5.89	6
	left	130.00	2.30	60.00	61.95	mono	11.21	−14.51	−7.92	
P4	right	160.00	3.50	90.00	359.27	mono	12.44	−14.41	−8.44	2
	left	160.00	3.00	60.00	147.44	mono	12.23	−14.01	−5.93	
P5	right	90.00	3.80	60.00	77.20	bi	10.18	−16.11	−8.85	6
	left	90.00	2.60	90.00	52.90	mono	10.68	−15.45	−9.15	
P6	right	210.00	4.70	60.00	243.09	mono	11.85	−12.94	−5.95	6
	left	210.00	4.60	90.00	465.57	mono	11.75	−12.67	−7.05	
P7	right	90.00	3.00	90.00	122.73	mono	12.67	−14.99	−5.69	2
	left	90.00	2.20	60.00	43.27	mono	12.00	−14.81	−6.50	
P8	right	160.00	2.40	90.00	86.22	mono	11.70	−14.47	−6.87	5
	left	160.00	3.90	90.00	269.73	mono	11.68	−13.61	−7.05	
P9	right	130.00	4.40	90.00	352.82	bi	11.50	−13.77	−8.00	1
	left	130.00	4.40	90.00	352.82	bi	12.35	−14.17	−7.35	
P10	right	130.00	2.80	60.00	84.23	mono	12.26	−12.87	−7.40	10
	left	130.00	3.50	60.00	149.06	mono	13.66	−13.07	−7.12	
P11	left	130.00	3.80	60.00	117.82	mono	10.58	−16.21	−6.76	2
	left	130.00	3.70	60.00	213.56	mono	10.54	−15.35	−8.73	
P12	right	90.00	3.80	60.00	169.88	mono	10.73	−14.50	−6.00	4
	left	90.00	3.80	60.00	155.95	mono	11.90	−14.13	−6.00	
P13	left	130.00	2.90	60.00	56.84	mono	12.86	−14.33	−7.56	1
	left	130.00	2.90	60.00	52.52	mono	12.59	−13.57	−5.34	
P14	right	130.00	2.90	60.00	85.64	mono	11.88	−13.97	−6.51	3
	left	130.00	2.90	60.00	85.64	mono	10.71	−13.42	−7.03	
P15	left	80.00	4.40	60.00	328.37	mono	11.29	−15.89	−8.15	7
	left	80.00	3.50	60.00	128.10	mono	11.18	−15.69	−8.92	
P16	left	130.00	1.30	60.00	29.89	mono	13.30	−12.60	−5.24	2
	left	130.00	1.70	60.00	39.07	mono	14.65	−12.18	−4.12	
P17	right	130.00	2.70	60.00	79.86	mono	11.11	−13.87	−7.78	4
	left	130.00	2.70	60.00	79.86	mono	12.31	−14.52	−7.07	
P18	right	130.00	2.10	60.00	65.77	bi	10.99	−13.59	−6.82	2
	left	130.00	3.50	60.00	112.68	mono	11.69	−14.08	−2.75	
P19	left	130.00	1.00	60.00	3.30	mono	11.42	−15.09	−8.29	1
	left	130.00	2.20	60.00	34.86	mono	11.93	−14.75	−8.55	
P20	left	130.00	0.50	60.00	0.94	mono	11.75	−14.16	−5.80	1
	left	130.00	0.50	60.00	0.79	mono	12.44	−14.02	−6.04	
P21	right	130.00	5.60	60.00	305.76	bi	12.00	−13.95	−3.45	6
	left	130.00	5.60	60.00	305.76	bi	12.38	−12.98	−4.10	

*bi*: bipolar; *mono*: monopolar.

Electrode coordinates correspond to the (mean values) of the active electrodes. Localisation data was not available for patient P1.

DBS electrode positions were determined using post-operative MRI. The image data were read into the standard Stereotactic Space from the Montreal Neurological Institute (MNI) [54], and atlas-specific coordinates were calculated for the active electrodes in each hemisphere (see Table 2).

### Verbal Fluency Task

Participants were asked to perform a VF task based on the German standard, the ‘Regensburger Wortfluessigkeitstest’/‘Regensburger Verbal Fluency Task’ [55] in which they had to utter as many German words as possible during a time period of 120 seconds under four task conditions: i) *semantic non alternating* (naming vegetables), ii) *phonemic non alternating* (naming words starting with ‘s’), iii) *semantic alternating* (naming animals and pieces of furniture alternatingly), and iv) *phonemic alternating* (naming words starting with ‘g’ and ‘r’ alternatingly). The order of the tasks was randomized for each participant. For the phonemic tasks they were explicitly encouraged to consider all lexical classes except for proper names. Repetitions of entire words or word stems were not allowed. The produced words were digitally recorded (computer software Audacity® 1.3.13-beta).

### Lexical Decision Task

In the LDT participants had to make word-nonword decisions upon auditory presentation of word-word or word-nonword sequences. At the beginning of each trial, a fixation cross appeared for 750 ms in the middle of a 17 inch computer screen, followed by a German noun (prime). 100 ms after the prime, either a real word or a pseudoword was presented. Primes and words/pseudowords were presented acoustically (individually adjusted volume via semi-open earphones; Beyerdynamic®, DT-880) eliciting several-fold larger effects and activating word processing more naturally than visual presentation [56], [57], cf. [22]. Real words were either only semantically related (*n* = 15), only phonemically related (*n* = 15) or semantically and phonemically unrelated (*n* = 15) to the prime word. Pseudowords were either phonemically related (*n* = 15) or unrelated (*n* = 30; since the occurrence of words and pseudowords should be equiprobable, unrelated pseudowords had to be as frequent as semantically related plus unrelated words). The participants were instructed to press a button as soon as they had identified a real word following the prime using their preferred hand, which was comfortably positioned over a push key. No response had to be given upon pseudowords.

The 90 trials from the different stimulus classes were presented in randomized order. Primes, words and pseudowords were never repeated. Reaction times (RT) were digitally logged by the used software (Presentation®, Version 15.0). For task repetition in the altered treatment ON or OFF conditions, a different set of stimuli was used. Participants performed practice runs of 10 trials that could be repeated until they felt familiar with the task.

Real words were mono- or disyllabic German nouns (mean duration 749±106 ms). Words were balanced for frequency as provided by the ‘Online-Wortschatz-Informationssystem Deutsch’/‘Online-Vocabulary-Information-System German’ (www.owid.de; mean frequency layer 8.3±1.3). Concerning relatedness, we built lists of related and unrelated word pairs since no comprehensive register of semantic relations exists for the German word pool. To confirm our definition of related versus unrelated words, the pairs were subsequently presented to 50 healthy adult native German speakers in random order (none of these subjects participated in the study later on) who rated the semantic relation on a 0–4 point scale (0 = no relation, 4 = highly related). Predefined *semantically related* words scored 3.5 (±.4), *unrelated* 1.1 (±.2) and *phonemically related* 1.3 (±.4) points. *T*-tests demonstrated significant differences between the relatedness scores of related and unrelated or phonemically related words (*p*<.01), whereas no difference was found between unrelated and phonemically related words. The phonemic relation between words (and pseudowords) was based on rhymes, i. e. only the initial consonants differed between the prime word and the target (pseudo-)word. Pseudowords were also mono- or disyllabic and resembled real words in that they were composed of existing German phonemes. The samples were recorded by a voice-trained female native German speaker (with a Zoom H4N® portable MP3/wave-recorder; 24 bit/96 kHz sampling rate). Word and pseudowords were cut and adjusted for volume (Audacity® 1.3.13-beta software).

### Statistical Analysis

#### Clinical and demographic data

We computed the total daily levodopa equivalence dose (LED) according to standardized conversion factors [58]. The total electrical energy delivered (TEED _1 sec_) was assessed as 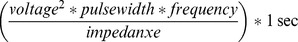 [59].

We used two-tailed t-tests for independent samples for group comparison of normally distributed data (*age*, *education*, *disease duration, LED* and *PANDA* score), the Mann-Whitney-*U*-Tests for non-parametric, non-dichtomous data (*Hoehn & Yahr)* and the χ^2^ test for dichotomous data (*gender, handedness* and *side of disease onset).* For comparing intraindividual score data between ON and OFF conditions, we used paired *T*-tests for dependent samples and, if normal distribution was not given, the Wilcoxon-signed-rank-test.

#### Verbal fluency task

Per task condition, we determined the total number of uttered words and errors (i. e. word and word stem repetitions, switch and category failures, names). To investigate group variation in task performance, we performed ANOVAs for the *total number of words*, for all groups (patients’ data from ON conditions). The analysis contained the within-subjects factor ‘task condition’ (4 levels: *semantic non-alternating, phonemic non-alternating*, *semantic alternating, phonemic alternating*) and the between-subjects factor ‘group’ (3 levels: *controls, non-DBS, DBS*). With respect to potential therapy effects, two further ANOVAs (DBS group/non-DBS group) were performed for the patients who participated in ON and OFF conditions using the same dependent variables. These two analyses each contained two within-subjects factors: ‘task condition’ (4 levels: *semantic non-alternating, phonemic non-alternating*, *semantic alternating, phonemic alternating*) and ‘therapy condition’ (2 levels: *ON, OFF*).

The statistical analysis of the percentage error rate (relative to the individual number of words) followed the ANOVA design detailed above.

#### Lexical decision task

RTs were determined from target to response onset (in ms). Outlier values were excluded, based on the Grubb’s test for outliers. Per subject, *mean RT* for each type of word relatedness (*semantically related, phonemically related, unrelated*) were calculated. The differences of RTs upon unrelated words versus either semantically or phonemically related words yielded the respective *Priming effects* (in ms).

Group differences in, *RT* and *Priming effects* were tested in two ANOVAs with the within-subjects factor ‘word relatedness’ (for *RT* 3 levels: *semantically related, phonemically related, unrelated/*for *Priming effects* 2 levels: *semantic/phonemic*) and the between-subjects factor ‘group’ (3 levels: *controls, non-DBS, DBS*). The same ANOVA approach as detailed above was used for assessing ON-OFF effects.

As error rate was not normally distributed, it was tested with non-parametric Wilcoxon (paired ON-OFF effects) and Mann-Whitney (unpaired group effects) tests. This was performed for the overall error rate as well as for the two types of false responses, namely error of commission (false hit to nonwords) and error of omission (no hit to word).

#### Additional calculations

Disease duration was on average three years longer in DBS than non-DBS patients (*p = *.07). To analyse if this influenced the results (cf. [60]), we built subgroups of long-term diseased patients (from the tenth disease year upwards) for non-DBS (*n* = 18, disease duration: 14.5±4.1 years) and DBS patients (*n* = 19, disease duration: 15.1±3.6 years; group difference n. s.: *p* = .64), and repeated the ANOVAs for VF and LDT for this constellation.

Since the DBS group contained significantly less female participants than the other groups, an explorative ANOVA with the between-subjects factors ‘gender’ was additionally run.

Further, we determined word articulation times (Audacity® 1.3.13-beta) to assess whether slowed articulation explained reduced word production rates in the VF task. Group differences were analysed using univariate ANOVAs (patients in the ON therapy condition). ON-OFF effects were assessed applying ANOVAs for repeated measures per treatment group.

#### Stepwise linear regression analysis

A stepwise linear regression analysis (SLR) is a statistical method for analysing which (out of numerous) variables provide the best explanation for the distribution of a dependent parameter. The approach starts with all candidate variables and, in an iterative procedure, those correlating least with the dependent parameter are removed until data prediction by the remaining ones becomes optimal. *P*-values for any of the thus determined variables express the likelihood of erroneously assuming a relation with the independent parameter.

We tested the variables *RT* and *Priming effect* from the LDT as well as baseline data from all participants, i. e. *age*, *netPANDA* (after subtraction of the test item VF), PANDA subscore for *working-memory*, *years of education* and *gender* against *VF* (the total number of uttered words) as the dependent parameter. Furthermore, *amplitude*, *frequency*, *pulse width*, *TEED*_1sec_ and *position of active electrode* (each separately for the right and left hemisphere) were tested as independent variables in the DBS group as well as LED in the non-DBS-group. All data pertaining to patient groups were taken from the treatment ON condition.

All statistical tests were performed with SPSS® version 19.0. For multiple comparisons Bonferroni corrections were applied.

## Results

### Clinical and Demographic Data

Controls and PD patients groups did not differ in age, handedness, and years of education. There was no significant difference in netPANDA, UPDRS motor scores, and side of disease onset between DBS and non-DBS patients in the respective ON therapy conditions. Due to reduced drug intake under stimulation, the LED was lower in DBS than non-DBS patients (see Table 1). The subgroups of patients who also participated in the treatment OFF conditions did not match in LED (non-DBS: 966.0±506.1 mg; DBS: 550.9±417.7 mg; *p*<.01), disease duration (non-DBS: 8.4±6.6; DBS: 13.8±4.3; *p*<.01), and UPDRS motor score (non-DBS: 27.7±12.5; DBS: 41.9±15.5; *p*<.01) in the OFF condition. The UPDRS motor score was significantly higher in the DBS OFF than DBS ON condition (40.6±16.2 vs. 20.0±7.2 points; *p*<.01). In the non-DBS group the increase in the UPDRS motor score after drug withdrawal (for 16.4±2.1 hs) was not significant (27.6±12.7 vs. 24.2±15.2; *p*>.05). NetPANDA scores were similar in both treatment ON versus OFF conditions (MED-ON vs. MED-OFF: 21.5±5.8 vs. 23.2±4.5; DBS ON vs. DBS OFF: 22.2±4.1 vs. 22.1±4; *p*>.05). Gender did not turn out to be a significant factor for task performance.

### Verbal Fluency Task

**Figure 1:**
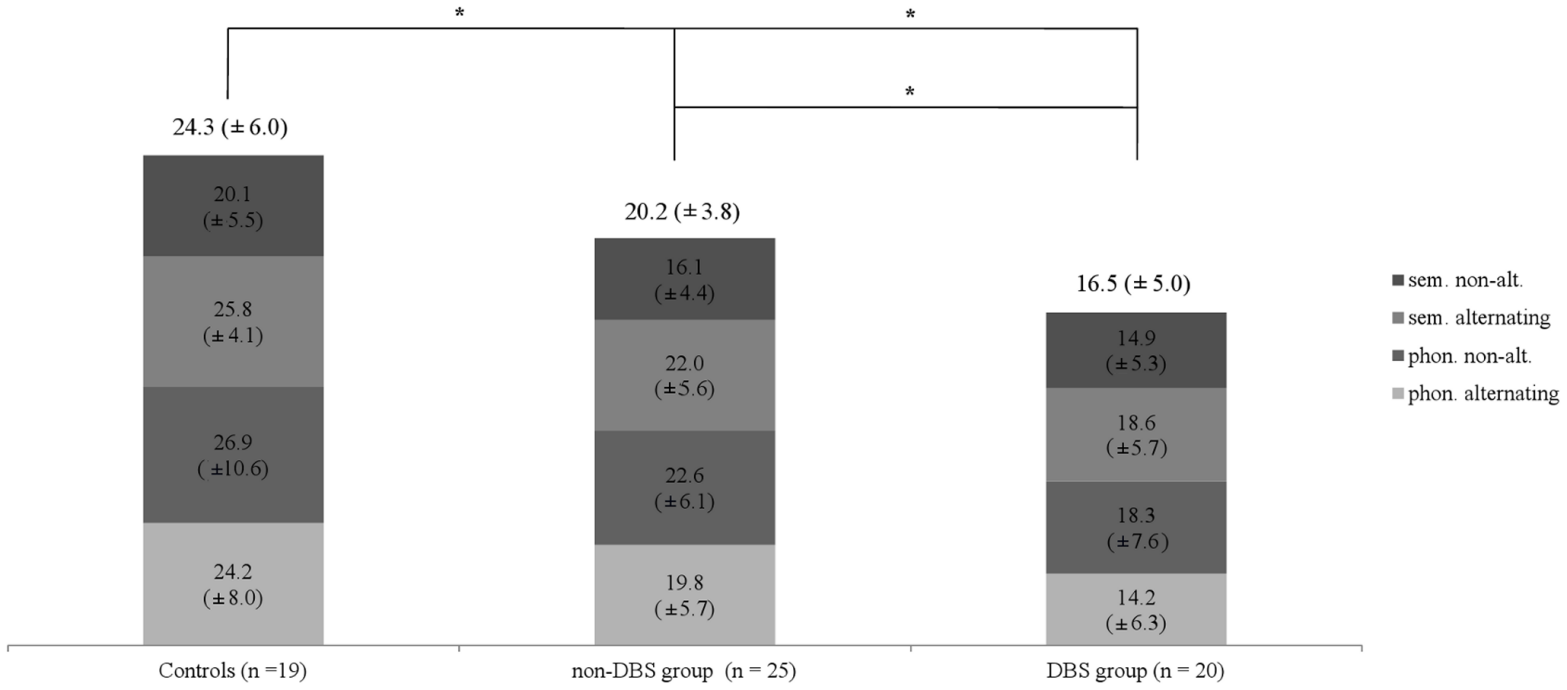
Verbal Fluency Task. The total number of words uttered by controls and both patient groups (each ON therapy) subdivided into the four task conditions; respective group average values are given on top of each column. Both patient groups produced significantly less words than controls. DBS patients uttered significantly less words than non-DBs patients. Values indicate the mean (± standard deviation), asterisks indicate statistically significant differnces (*: *p*<.05/**: *p*<.01). *sem.*: semantic; *phon.*: phonemic; *alt.*: alternating.

The first ANOVA comparing the total number of words between all three groups of participants indicated that ‘group’ (*F*_2,61_ = 12,2; *p*<.01) and ‘task condition’ (*F*_3_,_59_ = 24.3; *p*<.01) were significant factors. Post-hoc pairwise comparison revealed that controls uttered significantly more words than non-DBS (*p*<.05) and DBS patients (*p*<.05) who in turn performed worse than non-DBS patients (*p* = .05) (see Figure 1). No interaction was found. The same pattern and effect sizes were assessed for the subgroups of matched long-term DBS and non-DBS patients (main effects for ‘group’ [*F*_1,33_ = 6.0; *p*<.05] and ‘task condition’ [*F*_3,31_ = 9.6; *p*<.01], no interaction).

Separate ANOVAs for the non-DBS and DBS groups indicated no significant main effect of the ‘ON-OFF condition’ (non-DBS: *F*_1,12_ = 1.5; *p* = .2/DBS: *F*_1,15_ = .5; *p* = .5). As in the first ANOVA, ‘task condition’ was a main factor (non-DBS: *F*_3,10_ = 8.5; *p* = <.01/DBS: *F*_3,13_ = 18.0; *p*<.01) (see Table 3). No interactions were identified. The ANOVA for the error rate did not identify ‘group’ or ‘task condition’ as factors; no interaction was obtained. Neither medication nor stimulation influenced the error rate significantly.

**Table 3:** Verbal Fluency Task.

	non-DBS		DBS		*p-*values	
Words total	ON	OFF	ON	OFF	ON vs. OFF	
	M (± SD)	M (± SD)	M (± SD)	M (± SD)	non-DBS	DBS
mean	21.2 (±3.6)	19.6 (±3.9)	16.5 (±5.3)	16.1 (±5.3)	.49	.24
sem. alternating	23.1 (±7.1)	22.5 (±4.5)	18.3 (±5.9)	19.3 (±4.5)		
phon. alternating	20.8 (±3.6)	18.2 (±4.9)	14.3 (±6.7)	13.1 (±5.6)		
sem. non-alt.	17.1 (±4.2)	16.5 (±5.0)	15.2 (±5.5)	14.9 (±6.7)		
phon. non-alt.	23.8 (±4.5)	21.3 (±5.6)	18.1 (±8.0)	17.3 (±7.6)		

*sem*.: semantic; *phon*.: phonemic; *non-alt*: non-alternating.

The total number of words produced by non-DBS and DBS patients in the subgroups who participated in both the ON and OFF therapy condition is given for the subresults of the four task conditions and their mean; values indicate the mean (± standard deviation).

Concerning word articulation times, neither ‘group’ nor ^‘^ON-OFF condition’ were significant factors (controls:.86±.14 s, non-DBS ON/OFF:.79±.14 s/.76±.1 s; DBS ON/OFF:.78±.17 s/.80±.2 s).

### Lexical Decision Task

**Figure 2:**
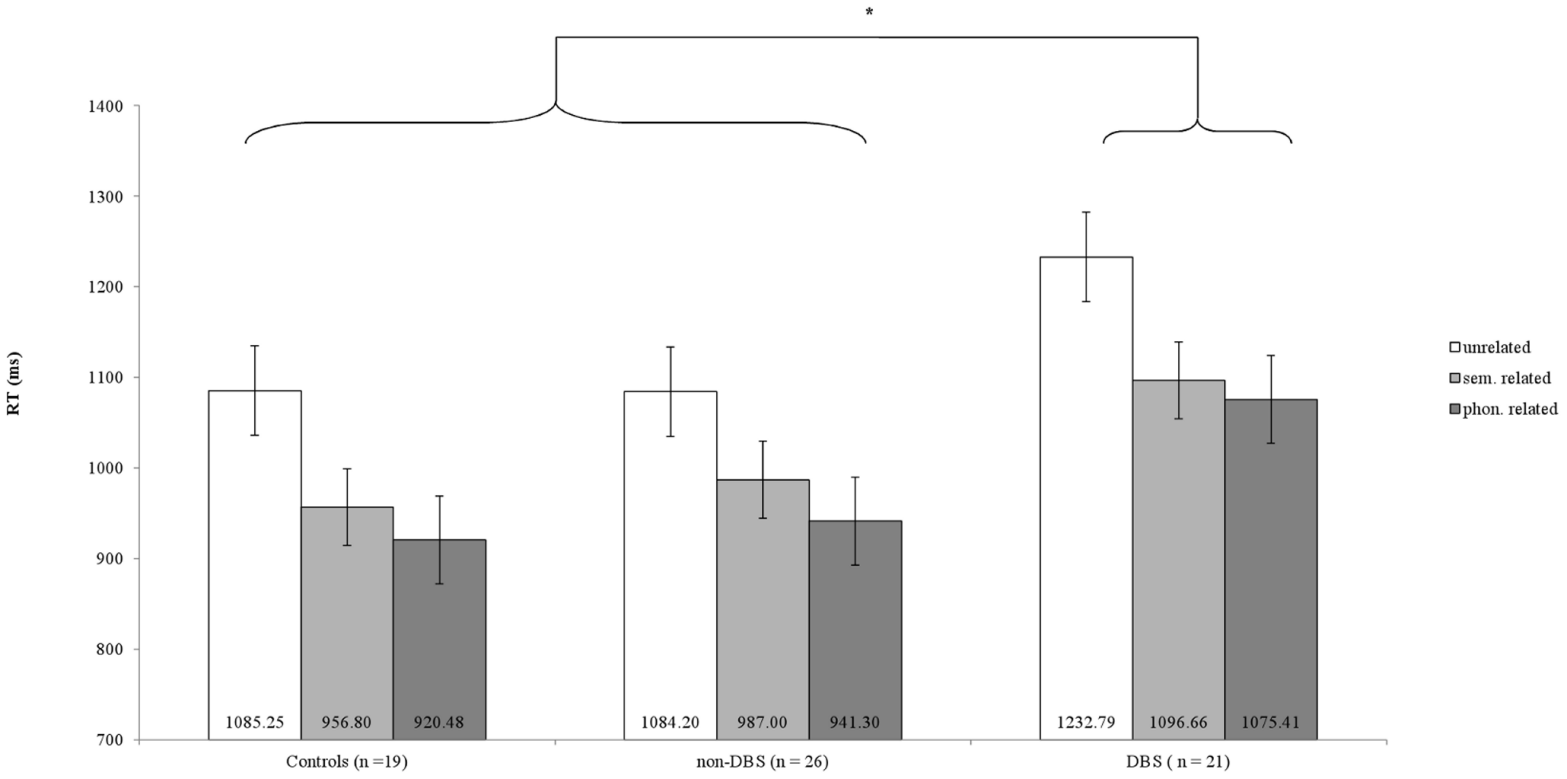
Lexical Decision Task. Reaction time in LDT indicated for the three stimulus conditions for controls and both patient groups (each ON therapy). DBS patients showed significantly longer RTs than controls and non-DBs patients. Shortest RTs were consistently obtained for phonemically related words. Values indicate the mean RT, bars the standard error, an asterisk a statistically significant differnces (*p*<.05).

In the ANOVA for *RT*, ‘group’ (*F*_2,63_ = 6.7; *p*<.01) and ‘task condition’ (*F*_2,62_ = 116; *p*<.01) were identified as main factors; no interaction was identified. We found longest RTs for *unrelated*, second longest for *semantically related* and shortest for *phonemically related words*. Accordingly, responses were accelerated more strongly by phonemic than semantic priming over all groups (mean *Priming effect*: phonemic = 153.8±87.7 ms, semantic = 118.6±79.2 ms; *p*<.01; see Figure 2). Post-hoc pairwise comparisons indicated that DBS patients reacted slower than controls (*p*<.01) and non-DBS patients (*p*<.01). The latter two groups behaved in essentially the same way (*p* = .1). The same held true when comparing only the groups of long-term diseased patients (‘group’: *F*_1,35_ = 5.6; *p*<.05; ‘task condition’ *F*_2,34_ = 62.8; *p*<.01).

In separate ANOVAS for the non-DBS and DBS group, ‘therapy condition’ was not a factor of *RT* or *Priming effect* (*RT*: non-DBS ON-OFF: *F*_1,13_ = .5; *p* = .3/DBS ON-OFF: *F*_1,18_ = 1.4; *p* = .2/*Priming*: non-DBS ON-OFF: *F*_1,13_ = 1.2; *p* = .3/DBS ON-OFF: *F*_1,18_ = 1.4; *p* = .3; see Table 4 for RTs). As in the first ANOVA, ‘task condition’ was a main factor of *RT* (non-DBS: *F*_2,12_ = 45.2; *p* = <.01/DBS ON-OFF: *F*_2,17_ = 47.6; *p* = <.01). Overall no differences in error rate were identified between groups. However, dividing into errors of commission (range from 5.5±6.3% to 10.6±6.1%) and errors of omission (range from 4.0±2.6% to 9.5±7.3%), it appeared that both patient groups made significantly more errors of commission in the treatment ON condition than controls (non-DBS: *p*<.05; DBS: *p*<.01) and that in the DBS group the rate of errors of omission significantly increased in the OFF condition (*p*<.05).

**Table 4:** Lexical Decision Task.

	non-DBS		DBS		*p-*values	
RT (ms)	ON	OFF	ON	OFF	ON vs. OFF	
	M (± SD)	M (± SD)	M (± SD)	M (± SD)	non-DBS	DBS
mean	1030.6 (±94.1)	1048.4 (±115.8)	1143.6 (±225.6)	1183.2 (±272.6)	.48	.29
unrelated	1098.3 (±88.8)	1133.4 (±118.6)	1241.8 (±243.9)	1299.7 (±270.2)		
sem. related	1022.7 (±120.5)	1027.5 (±105.5)	1102.0 (±220.9)	1137.3 (±269.3)		
phon. related	974.0 (±96.3)	987.4 (±143.1)	1090.6 (±227.3)	1125.7 (±297.5)		

*RT*: reaction time; *sem.*: semantic; *phon.*: phonemic.

Reaction time in LDT of the subgroups of non-DBS and DBS patients who participated in both the ON and OFF therapy condition are presented for the three distinct stimulus conditions and their mean. Values indicate the mean (± standard deviation).

### Stepwise Linear Regression Analysis

SLR yielded a significant model (*R* = .47; constant = 105.64; *F*_2,63_ = 8.75; <.001) with two coefficients: 1) *RT (b1* = −.064, beta standardized = −.43; *p*<.001) and 2) *years of education* (*b2* = 3.37 beta standardized = .26, p = .028). No significant correlation between VF results and any of the other parameters tested was identified.

## Discussion

In patients with STN-DBS, VF and LDT performances were abnormal both compared to healthy subjects and to non-DBS PD patients. In VF tasks, word production was low, regardless of the task condition. In the LDT, response latencies were prolonged whether words were primed or not. Non-DBS PD patients performed worse than controls only in VF, but not in the LDT. Neither the medication nor the DBS ON-OFF condition led to significant changes in task results. Low VF performance could not be explained by slowed speech rates which could have been due to DBS current spread to corticobulbar or cerebello-thalamic fibres [4], since word articulation times did not differ between groups or treatment conditions. Furthermore, no evidence was found that prolonged RTs were due to worse motor conditions in DBS patients, as their UPDRS motor scores were lower than those of non-DBS patients.

Different cognitive explanations for the results are conceivable. For instance, a disturbance of specific verbal processes could cause perturbation of both VF and LDT performances. But, of note, priming effects in the LDT were preserved across all study groups and treatment conditions. From this it seems that functions of the genuine semantic and phonemic network activation [47] were not affected by the disease or the therapies applied. Also, the overall task accuracy was unaltered. Thus, the results in the DBS group might rather point to a problem of general cognitive slowing, while leaving qualitative characteristics of lexical processing intact. Such a mechanism also appears in line with the moderate but highly significant correlation between RTs in the LDT and the number of words produced in VF tasks.

VF had not been assessed in parallel with response latencies before. But in a few studies relations between VF and mental speed have been suggested based on reduced performance in Trail-Making or Stroop tasks [6], [7], [17]. Since in the current study both tasks specifically addressed lexical operations, future chronometric studies might examine connections between mental speed and non-lexical cognitive domains.

Importantly, the slowing in the DBS patients was irrespective of the stimulation state. Further, it was independent from disease duration and - in line with most previous findings [5], [25]–[27], [30] – no correlation between task performances and stimulation parameters was identified. This constellation is well compatible with the assumption that the results in DBS patients reflect sequels of the surgical intervention for the therapy. In this context it is of note that recently the passage of DBS lead trajectories through the head of the caudate nucleus has been suggested to underlie postoperative cognitive decline [19]. Having said this, it is important to mention that VF unresponsiveness to STN neuromodulation is true for the given stimulator settings and electrode positions, but that target-related effects cannot be generally dismissed. For example, postoperative VF decline has been associated with relatively ventral electrode positions corresponding to limbic and associative segments of the STN [19], and a gradient from decreased to increased VF has been proposed from medial to dorsolateral STN-DBS [28], [29]. Besides, VF improvement has been described for experimental low frequency stimulation at 10 Hz [61] and, in another study, VF worsening by therapeutic stimulation [18].

With respect to concepts of subcortical language processing, the results are compatible with models which do not assume the basal ganglia to be specifically involved in lexical operations [39]–[43]. Regarding concepts of general cognitive functions of the STN, one subresult concerning task accuracy shall be mentioned. Although the overall error rate in the LDT was unaffected, patients in the treatment ON condition showed a somewhat higher rate of errors of commission (hits upon non-words), whereas the DBS OFF condition led to increased errors of omission (no response to words). This pattern seems in line with proposed STN functions in response selection and their modulation by DBS [60], [62], [63].

Comparable with other studies [24], [60], VF was also low in the non-DBS group, though to a lesser extent. The complete independence from the medication status suggests a disease-related origin of this deficit.

In conclusion, a history of subthalamic DBS surgery appears to exacerbate VF deficits in PD and to slow down performance in the LDT despite massively improving the motor condition. A reduction of mental speed is a candidate mechanism for these effects which should be further tested in non-lexical cognitive tasks. The slowing was not modified by the proper stimulation of the DBS target region. Further studies might therefore focus on possible relations between cognitive speed and the structures passed through by the surgical trajectory in order to further refine the procedure.
